# Dental Convention

**Published:** 1856-12

**Authors:** 


[For the Dental Register.]

That associations of members of the dental profession for mutual improvement are highly advantageous, no one will deny. With the proceedings of the late American Dental Convention, the report of which I read for the first time last evening, I am pleased; while its selection of place of holding its next meeting is, in my humble opinion, not judicious.

The Convention should be a great *national* gathering of the profession, where our strong men freely impart from their stock of knowledge for the benefit of all; and its controling spirit should be free from sectionalism, or partiality. No one attends such meetings without adding to his acquirements, and feeling more pride in his noble profession, as well as a greater ambition to make advances in his attainments. Thus the investment of time and money for this purpose is amply repaid.

Locating the next Convention at Boston is decidedly an eastern affair, to say the least of it. The Convention having held its meeting last year at Philadelphia, and the present year at New York, was it not proper its next session should be held west of the Alleghanies, where are about half the dentists of the nation? Did not the attendance of western dentists, including three of the six professors in the Ohio College of Dental Surgery, and a good number of other distinguished operators, notwithstanding their long line of travel to reach it, evince great interest in the Convention? It appears from the report that they acquitted themselves in a manner worthy of the noble region they represented.

What claims had Boston as a place for holding the next session of the Convention? How many of her more than an hundred dentists felt sufficient interest to induce them to make the trip of about eight or ten hours to be present at that meeting? Just two, all told. What have our Boston brethren ever done to promote the interests of the profession —what improvements have they communicated—what efforts to elevate the standard of dental education—what associations among themselves—what contributions from their pens for the dental journals? Echo answers. And yet, regardless of the claims of all the country west of Philadelphia, the next Convention must be held at Boston, almost on the eastern verge of our territory. And why? To do good to be sure, to wake up the natives, and to get them to work for the general good of the profession. But would not much greater beneficial results follow the holding the Convention at the west? Unless the profession in Boston are prepared in their hearts for efforts different from those they have heretofore made, the Convention, as it possesses no regenerating power, could at most only get up a spasmodic dental revival, the effects of which would be evanescent. But in the west, among dentists full of the go-ahead spirit, a session of the Convention would no doubt be productive of permanently good results.

But the hope of having it there must be deferred for several years; and even then it is not probable its originators would willingly have a session of it held there oftener than once in a half dozen years.

In this state of affairs is it not advisable to hold annual conventions, with a broad platform, open to all who have been practising dentists, at the most important points in the vast field west of the Alleghanies? There is a work for such a convention, which the Mississippi Valley and the Western Dental Associations do not reach; and which would be harmonious with theirs. We have all the elements for success in such an enterprise; with our lakes, rivers, and immense net-work of railways, almost every dentist throughout this vast region could, with little trouble, reach any locality where the convention might be held.

I do not propose this in opposition to the American Convention—let as many western dentists as possible attend its meetings—but there is great need of such an *annual* convention in the western field.

Please let us have a free expression of opinion on the subject in the next Register. Why not get up such a convention next summer at St. Louis, Louisville, Cincinnati, or some other point? A Yankee.

Nov. 3, 1856.

